# Identification and Characterization of circRNA in Longissimus Dorsi of Different Breeds of Cattle

**DOI:** 10.3389/fgene.2020.565085

**Published:** 2020-11-26

**Authors:** Ruili Liu, Xianxun Liu, Xuejin Bai, Chaozhu Xiao, Yajuan Dong

**Affiliations:** ^1^Laboratory of Animal Physiology and Biochemistry, Animal Embryo Center, College of Animal Science, Qingdao Agricultural University, Qingdao, China; ^2^Laboratory of Animal Molecular Shandong Black Cattle Breeding Engineering Technology Center, College of Animal Science, Qingdao Agricultural University, Qingdao, China

**Keywords:** Luxi cattle, circRNA, skeletal muscle, Shandong black cattle, identification

## Abstract

Shandong black cattle is a new breed of cattle that is developed by applying modern biotechnology, such as somatic cloning, and conventional breeding methods to Luxi cattle. It is very important to study the function and regulatory mechanism of circRNAs in muscle differentiation among different breeds to improve meat quality and meat production performance and to provide new ideas for beef cattle meat quality improvements and new breed development. Therefore, the goal of this study was to sequence and identify circRNAs in muscle tissues of different breeds of cattle. We used RNA-seq to identify circRNAs in the muscles of two breeds of cattle (Shandong black and Luxi). We identified 14,640 circRNAs and found 655 differentially expressed circRNAs. We also analyzed the classification and characteristics of circRNAs in muscle tissue. Gene Ontology and Kyoto Encyclopedia of Genes and Genomes analyses were used on the parental genes of circRNAs. They were mainly involved in a variety of biological processes, such as muscle fiber development, smooth muscle cell proliferation, bone system morphogenesis, tight junctions and the MAPK, AMPK, and mTOR signaling pathways. In addition, we used miRanda to predict the interactions between 14 circRNAs and 11 miRNAs. Based on the above assays, we identified circRNAs (circ0001048, circ0001103, circ0001159, circ0003719, circ0003424, circ0003721, circ0003720, circ0001519, circ0001530, circ0005011, circ0014518, circ0000181, circ0000190, circ0010558) that may play important roles in the regulation of muscle growth and development. Using real-time quantitative PCR, 14 circRNAs were randomly selected to verify the real circRNAs. Luciferase reporter gene system was used to verify the binding site of miR-1 in circ0014518. Our results provide more information about circRNAs regulating muscle development in different breeds of cattle and lay a solid foundation for future experiments.

## Introduction

CircRNA is a unique kind of non-coding RNA that has no 5′ terminal cap or 3′ terminal poly (a) tail and presents a closed ring structure (Rong et al., 2017). Kolakofsky (1976) and Sanger et al. (1976) first discovered circular RNAs (showed closed loop) in plant viroid and parainfluenza virus particles by electron microscopy. It was found that most of the circRNAs originate from exons and a few from introns. In terms of function, circRNAs mainly adsorb miRNAs through the “molecular sponge” mechanism, thus inhibiting the regulatory role of miRNAs and enhancing the expression levels of target genes (Memczak et al., 2013; Traurig et al., 2013; Zhang et al., 2016). In recent years, circRNA has become a new research hotspot in the field of scientific research. It has been found that circRNAs are involved in many biological processes, including growth and development, and diseases, among others. Ivano sequenced human and mouse myoblasts and compared the expression of circRNAs in myoblasts and myoblasts of Duchenne muscular dystrophy. It was the first time that circRNA was expressed during myogenic differentiation. Thirty one significantly different circRNAs were screened out, which laid a foundation for the study of circRNA in skeletal muscle development (Legnini et al., 2017). Subsequent studies found that circRNA was expressed in the skeletal muscle of pigs, cattle and other animals, and its expression was spatiotemporal and interspecific. Li found that the circRNAs circFUT1O and circFGFR4 could regulate the proliferation and differentiation of bovine skeletal muscle cells by absorbing miR-133a and miR-107, respectively (Li et al., 2017, 2018). Wei found that circLMO7 can regulate the differentiation and apoptosis of bovine skeletal muscle cells by absorbing miR-378a-3p (Wei et al., 2017). In conclusion, circRNAs play an important role in the development of bovine muscle. There are almost no reports about the regulation by circRNAs of the development of bovine skeletal muscle. In addition, we found that the current research on circRNAs is mainly focused on cancer, while the research on livestock and poultry mainly focuses on different periods (fetal vs adult), and there is little research on different varieties. This study is mainly about identification and characterization of circRNA in different breeds of cattle, which has certain theoretical significance for breeding.

Shandong black cattle are the first embryo transfer calves in China and are obtained by vitrified frozen somatic cell cloning embryos. According to the breeding goal, the new generation core breeding group was established by filial generation of Shandong black cattle. Then the new generation core breeding group hybridized with Shandong black cattle. Then the ideal black cattle and Shandong black cattle were selected from the second generation group and entered the cross-cross fixation stage. After four generations, it was finally bred into a new Shandong black cattle variety matching line and used as a bull. After careful cultivation by researchers, Shandong black cattle bulls were used in breeding. At present, there are 351 breeding bulls and 694 basic cows that meet the requirements of breeding objectives. Their average weight is 920 kg bulls and 510 kg cows. In 2015, Shandong black cattle was approved by the National Animal and Poultry Genetic Resources Committee as a new population and successfully established a new variety cultivation Chinese base. With the approval of the local government, we have established the local standard of Shandong black cattle. The major genes CAST, MSTN, and CAPN1 related to fat deposition, muscle tenderness and marbling have been preliminarily screened, As a molecular marker for early screening of cattle, ultrasonic was used to determine marbling, eye muscle area, back fat thickness, and intramuscular fat content. The purpose of this study was to investigate the identification and characterization of cirRNA in Hybrid Progenies (Shandong black cattle) and primordial maternal generation (Luxi cattle), and further explore its regulation related to production performance. Finally, high-quality beef cattle were selected to realize the leap from new varieties to new varieties.

We selected Shandong black cattle and Luxi cattle for this research study, identified circRNAs in muscle tissue, analyzed their genomic characteristics, expression differences, etc. Through high-throughput sequencing technology combined with functional verification tests, we revealed the function and regulatory mechanism of circRNA in muscle development and combined these findings with the characteristics of different breeds to cultivate fast-growing and high meat production rate beef cattle varieties to increase the speed of beef cattle breeding and provide a theoretical basis for the development of China’s beef industry.

## Materials and Methods

### Sample Preparation

The animals used in this research institute, Shandong black cattle and Luxi cattle, were selected from Shandong black Cattle Technology Co., Ltd., and Dadi yellow cattle. Three healthy Shandong black cattle and three Luxi cattle animals at 18 months old were selected. All experimental cattle were raised in the same farm environment, and the samples were stored in liquid nitrogen to extract total RNA. Muscle tissue samples were fixed in 4% formaldehyde and stained with hematoxylin and eosin (HE) for histological observation. All experimental and surgical procedures involved in this study followed the “guidelines for experimental animals” of the Ministry of Science and Technology (Beijing, China). All procedures and animal care were in line with the recommendations of the European Commission (1997) and were approved by the Experimental Animal Ethics Committee of Qingdao Agricultural University.

### HE Staining of Muscle Tissue

Paraffin sections were made from muscle tissue fixed with 4% paraformaldehyde. The HE staining protocol included dewaxing, covering with water, hematoxylin staining, washing with water, 5% acetic acid differentiation, back blue, eosin staining, dehydration, natural drying, sealing, and image acquisition. The specific steps have been described previously (Cao et al., 2014). HE staining images were used for counting, and the surface area was measured by Image-Pro Plus software. SPASS software was used for statistical analysis to calculate significant differences.

### circRNA Sequencing

High-throughput full transcriptome sequencing and subsequent bioinformatics analysis were carried out by Annoload Technologies (Beijing, China) as follows. Total RNA was extracted from the longissimus dorsi muscle of Shandong black cattle (B) and Luxi cattle (L) using TRIzol reagent (TIANGEN) according to the manufacturer’s instructions, and genomic DNA was removed with DNase I (Takara). RNA quality (RNA integrity number, RIN) was determined by an Agilent 2100 Bioanalyzer, and ND-2000 (NanoDrop) was quantified. Using a Ribo-Zero Gold Kit to remove rRNA from samples, and according to the specifications for the NEBNext Ultra Directional RNA Library Preparation Kit for Illumina (NEB, Ipswich, MA, USA), different index tags were selected to construct the library, and Illumina was used to sequence the constructed library.

### Read Quality Control and Mapping

The original paired-end reads were trimmed and filtered for quality Trimmomatic using the default parameters.^1^ Then, TopHat software^2^ was used to align the clean reads with the reference bovine genome^3^ and obtain the orientation pattern. The software was used to align the RNA sequence reads with the genome to detect gene expression and exon splicing. The genome was constructed on the superfast short read mapper Bowtie 2 for mapping with default parameters.

### Identification of Differentially Expressed circRNAs

We used SRPBM [spliced reads per Billion Mapping, defined as number of circular reads/(number of mapped reads × read length)] as a standardized method to quantify the expression of circRNA, and DEseq2 was used to analyze the differential expression of circRNA (Love et al., 2014). In pairwise comparisons, circRNAs with *P* < 0.05 and absolute fold change value greater than 1.5 were considered to be significantly differentially expressed, and finally, the number of upregulated and downregulated circRNAs was obtained.

### Enrichment Analysis of GO, KEGG, and PPI Pathways

Gene Ontology (GO) analysis and KEGG pathway analysis of the parental genes of differentially expressed circRNAs were used for annotation. The Blast2GO method was used for GO function analysis. KOBAS software was used to test the statistical enrichment of differential gene expression in the KEGG pathway. When *P* < 0.05, GO terms and KEGG pathways were considered to be significantly enriched.

### Prediction of miRNA Targets of circRNAs

To explore the function of circRNAs and predict which circRNA acts as a miRNA sponge, we used miRanda (3.3a)^4^ to predict the target relationship (John et al., 2004). In view of the published reports and the extractability of the sequences, we only selected the type of classical (when the formation site of the circRNA was exactly on the boundaries of exons) and antisense (when the circRNA was formed by the antisense strand of the gene) circRNAs to predict the miRNA targeting relationship.

### Experimental Verification of circRNA

Real-time quantitative PCR (qRT-PCR) was used to verify the expression of circRNA. The expression levels of the selected circRNAs were standardized with the levels of the housekeeping gene GAPDH. Primer 3.0 software was used to design primers (Supplementary Table 7), which were synthesized by SANGON Biotechnology Co., Ltd. (Shanghai, China). Total RNA was converted into cDNA using random hexamers with Transcriptor First Strand cDNA Synthesis Kit (Roche, Australia). The qRT-PCR reaction (20 μL) consisted of 1 μL template cDNA, 5 ml (5 × 1 ml vials) 10 μL upstream and downstream primers, respectively, RNAse-free water. The procedure was as follows: 94°C for 10 min and 40 cycles of 94°C for 30 s, 60°C for 30 s, and 72°C for 40 s. The expression of circRNAs were calculated by the 2^–ΔΔ^ ^*CT*^ value method.

### Luciferase Reporter Assay

Cells were seeded in 96-well plates at a density of 5 × 10^3^ cells (HEK-293T) per well 24 h before transfection. The cells were co-transfected with a mixture of 50 ng FL reporter vectors, 5 ng Renilla luciferase (RL) reporter vectors (pRL-TK), and miRNA mimics at the indicated concentration. The miRNA mimic were obtained from Life Technologies. After 48 h, the luciferase activity was measured with a dual luciferase reporter assay system (Promega, Madison, WI, United States). In this study, the double luciferase vector psi check-2 was selected, and the bta-circ0014629 sequence was cloned into the reporter gene vector psicheck to synthesize the predicted miRNA mimics and control. MiRNA mimics and psicheck-bta-circ0014629 were transfected into 293T cells at the same time. The expression level of reporter genes was detected by multi-functional enzyme labeling instrument, and the miRNAs with down-regulation of reporter gene expression were screened. It is necessary to clone the potential binding target of miRNA into the 3′UTR region of r-luciferase (hrluc), and then co transfect with the miRNA to determine the activity of R-Luciferase. F-Luciferase (hluc+) is used as the internal reference to correct the transfection efficiency between different samples.

## Results

### Apparent Differences in Muscle Fibers in Different Breeds of Beef Cattle

**FIGURE 1:**
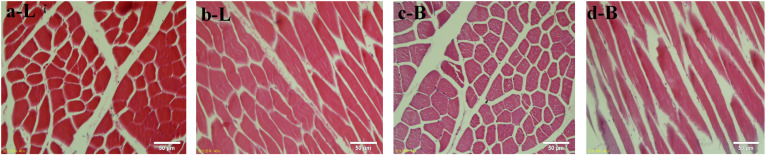
HE staining of muscle tissue paraffin sections. Red represents a single muscle fiber, blue represents a nucleus (marked by arrows); a and c stand for tissue crosscutting, b and d stand for tissue longitudinal cutting; L stands for Luxi cattle, B stands for Shandong black cattle.

The muscle fibers of the longissimus dorsi in the back of Shandong black cattle and Luxi cattle were significantly different in the apparent observation of paraffin sections stained by HE (Figure 1). The length of single muscle fibers of Shandong black cattle was significantly longer than that of Luxi cattle, and the number of nucleus in each muscle fiber was also greater. The boundary between the muscle fibers of Shandong black cattle was clearer and rounder than that of Luxi cattle. IPP software analysis showed that there were significant differences in the muscle fiber diameter, length and weight (*P* < 0.05) but no significant differences in other muscle fiber properties (*P* > 0.05) (Table 1).

**TABLE 1:** Comparison of muscle fiber characteristics and growth characteristics of different breeds of cattle.

Characteristics	Luxi cattle	Shandong black cattle
Area (μm^2^)	4831.21 ± 255.314	5224.373 ± 442.365
Diameter (μm)	59.339 ± 0.944*	65.473 ± 2.054
Length (μm)	106.722 ± 8.306*	165.965 ± 8.874
Density (number of muscle fibers/muscle fiber area, EA/μm^2^)	1543.777 ± 61.880	1744.158 ± 102.999
Number of muscle fibers (EA)	157.125 ± 5.959	139.833 ± 7.0618
Weight (kg)	291.063 ± 3.335*	309.500 ± 4.992

*Asterisk indicates a significant difference (P < 0.05).*

### Transcriptome Quantification

To examine the expression profile of circRNAs in the longissimus dorsi muscle of different breeds of beef cattle, we established a cDNA library of six longissimus dorsi muscle samples from Luxi cattle (L) and Shandong black cattle (B) and obtained the raw data after Illumina sequencing. After removing the low-quality reads and the reads with adapter sequences from the RNA-seq raw reads, 223,609,230 clean reads (B: 122,463,284, L: 101,145,946; Table 2) were obtained. Then, we queried the clean reads of the latest reference genome and used TopHat (see text footnote 2) for mapping. In the B and L samples, 98.08 and 97.53% of the reads were located in the reference genome, and the clean (Q30) base rates were 94.30 and 93.72%, respectively.

**TABLE 2:** Read quality and mapping results for RNA-seq.

Sample	Total raw reads	Total clean reads	Mapped reads	Mapping rate (%)	Clean (Q30) base rate (%)
L1	126,735,736	122,463,284	120,167,801	98.13	94.00
L2	125,664,524	119,811,368	117,518,654	98.09	94.36
L3	125,900,178	119,637,778	117,281,323	98.03	94.54
B1	99,182,298	95,960,692	94,094,172	98.05	93.75
B2	105,345,396	101,145,946	97,711,021	96.60	93.56
B3	102,435,924	98,666,436	96,633,740	97.94	93.86

### DEG Analysis and Functional Annotation

**FIGURE 2:**
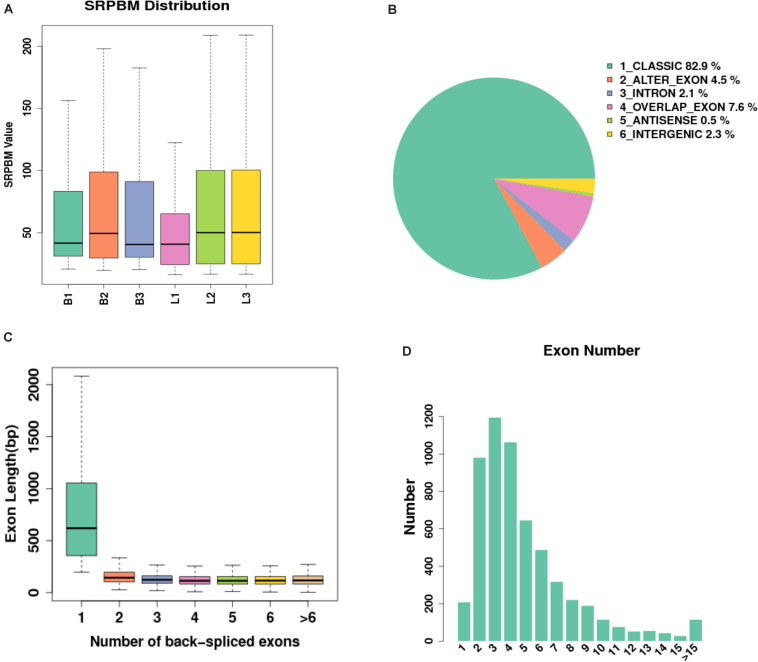
Expression level of sample circRNAs. **(A)** SRPBM density: distribution density of circRNA expression in the sample; SRPBM value: the normalized count value of circRNAs, calculated by a formula [SRPBM = (SR×10^9^)/N] in which SR is the number of spliced reads, and N is the total number of mapped reads in samples. **(B)** Distribution pie chart of the six types of circRNAs; **(C)** Box plot of the single exon length distribution of circular RNAs. The abscissa represents the number of exons contained in the circRNA, and the ordinate represents the length of a single exon; **(D)** The distribution of the number of exons contained in circular RNAs in the sample, the abscissa represents the number of exons, and the ordinate represents the number of circRNAs corresponding to the number of exons.

In this study, 14640 circRNAs and 4201 parental genes (Supplementary Table 1) were detected, of which 1591 (10.86%) were expressed in all samples (Figure 2A). The SRPBM values (one billionth of the splicing reading) were used to calculate the expression levels of circRNAs. We list the 30 highest expression circRNAs in each group (Table 3). According to their position in the genome, 14856 circRNAs were divided into six types: classical: when the formation site of the circRNA was exactly on the boundaries of exons (82.9%), alter exon: when one end of the circRNA formation site was on the exon boundary, and the other end was inside the exon (4.5%), intron: when the formation site of the circRNA was completely in the intron region (2.1%), overlap exon: when the formation site of the circRNA spanned the exon region (7.6%), antisense: when the circRNA was formed by the antisense strand of the gene (0.5%), and intergenic: when the formation site of circRNA was completely inside the intergenic region (2.3%) (Figure 2B). In a single exon, the length of the exon was significantly longer than that of the circular RNA composed of multiple exons (Figure 2C). circRNAs were mainly encoded by two to four exons (Figure 2D).

**TABLE 3:** The 30 circRNAs with the highest expression in each group.

B-circRNA_ID	B-source_gene	L-circRNA_ID	L-source_gene
circ0013465	gene 30421	circ0013465	gene 30421
circ0011592	gene 25926	circ0011592	gene 25926
circ0001880	gene 2182	circ0001880	gene 2182
circ0008118	gene 16072	circ0006451	gene 12913
circ0012379	Null	circ0012379	Null
circ0006451	gene 12913	circ0005801	gene 12000
circ0010250	gene 21361	circ0012393	Null
circ0011321	gene 25624	circ0005239	gene 11048
circ0003768	gene 6914	circ0008118	gene 16072
circ0000295	gene 1312	circ0011321	gene 25624
circ0005801	gene 12000	circ0012381	Null
circ0005239	gene 11048	circ0014578	gene 33995
circ0012381	Null	circ0003768	gene 6914
circ0007282	gene 14472	circ0000295	gene 1312
circ0012244	gene 27622	circ0000448	gene 267
circ0003777	gene 6914	circ0012335	gene 27977
circ0000923	gene 2584	circ0005452	gene 11270
circ0013776	gene 31637	circ0013776	gene 31637
circ0009975	gene 20815	circ0002476	gene 2820
circ0000448	gene 267	circ0005082	gene 10664
circ0002476	gene 2820	circ0012389	Null
circ0007065	gene 14054	circ0000923	gene 2584
circ0008388	gene 16629	circ0009975	gene 20815
circ0003798	gene 6918	circ0008388	gene 16629
circ0001875	gene 2179	circ0008946	gene 17970
circ0001112	gene 1570	circ0009532	gene 19466
circ0008946	gene 17970	circ0001205	gene 1570
circ0009481	gene 19383	circ0001212	gene 1570
circ0009532	gene 19466	circ0006938	gene 13857
circ0010865	Null	circ0001083	gene 1570

### Differential Expression of circRNAs

**FIGURE 3:**
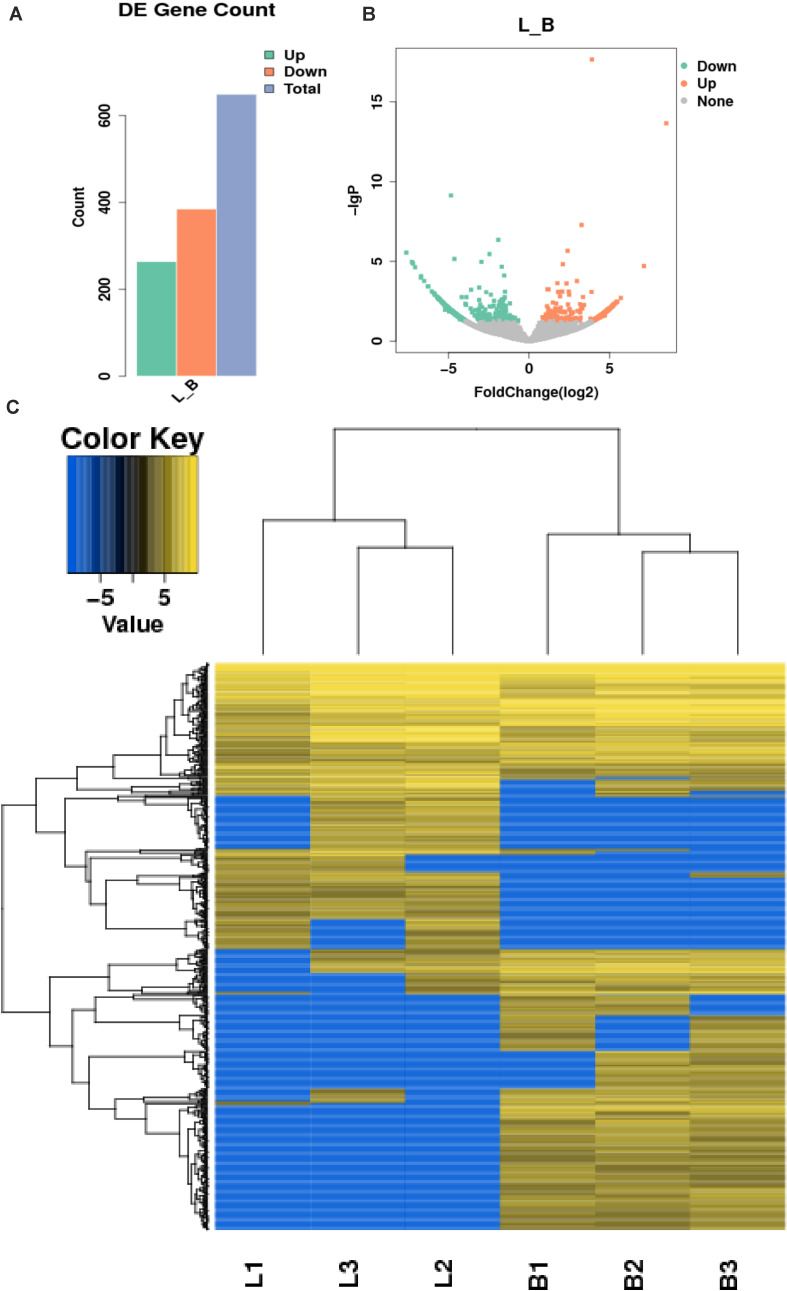
Differential expression of circRNAs in the samples. **(A)** Statistical chart of the differential expression of 655 DE circRNAs, of which 267 were upregulated and 388 were downregulated; **(B)** Volcanic chart of differential circRNAs, the abscissa is the fold change of expression in L and B samples, the ordinate is the statistical significance of the change of expression. Different colors indicate different classifications: green (downregulated), orange (upregulated), and gray (the difference is not obvious); **(C)** Cluster heat map of differentially expressed genes. The change in expression quantity is represented by the change in color, blue represents lower expression levels, and yellow represents higher expression levels.

In this study, 655 differential circRNAs and 467 parental genes (Supplementary Table 2) were detected, of which 267 were upregulated and 388 were downregulated (Figure 3A). circRNAs with significant differential expression are visualized by a red volcano map (Figure 3B) and cluster heat map (Figure 3C) to illustrate the distribution of differential circRNAs.

### Enrichment Analysis by GO, KEGG, and SPONG

**FIGURE 4:**
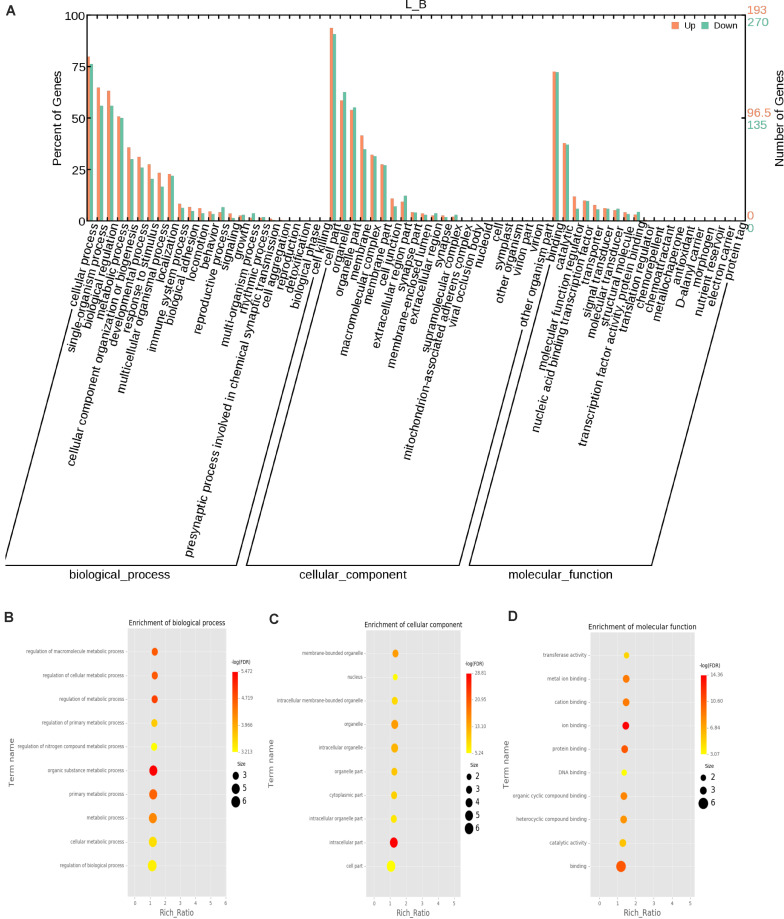
GO annotation of differential circRNAs and enrichment analysis. **(A)** GO annotation of the parental genes of DE circRNAs. The abscissa is the ontology classification, and the ordinate is the proportion of genes annotated to this term among all annotated genes; **(B–D)** *Q*-value enrichment map of single group GO entries. Each point represents the estimated enrichment degree of the corresponding GO term, and the closer the color is to red, the higher the enrichment degree. The size of each point indicates the number of genes enriched in the GO term, and the larger the point is, the more genes enriched in the GO term, and vice versa.

**FIGURE 5:**
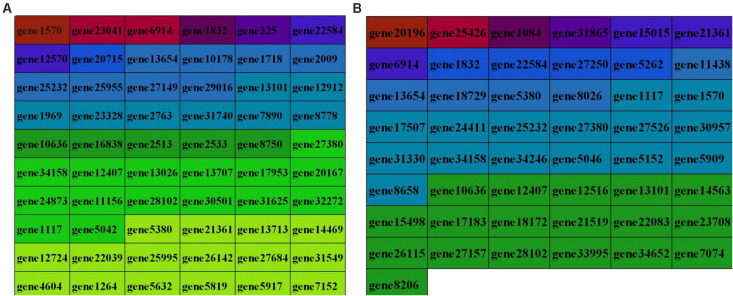
Enrichment degree of the identified parental genes in GO terms **(A)** and KEGG pathways **(B)**. Different colors indicate the different enrichment degrees of parental genes in terms related to muscle growth and development. The darker the color, the higher the enrichment degree and the lighter the color, the lower the enrichment degree; the enrichment degree of source genes is arranged from high at the top to low at the bottom.

The function of a circRNA is reflected by its parental genes. To determine the function of these genes, GO analysis was carried out. According to the statistical data of differentially expressed circRNAs and their parental genes, we annotated the parental genes of circRNAs instead of the circRNAs because there is currently no information on the annotation of circRNAs. The parental genes of differentially expressed circRNAs were annotated by 65 different GO terms (Figure 4A). The most annotated GO terms were cellular process (BP), single organization process (BP), cell part (CC), organelle (CC), binding (MF), and catalytic (MF). To understand the functions of DEGs, goatools^5^ were used to conduct functional enrichment GO analyses. The results showed that 142 differentially expressed parental genes of circRNAs were significantly enriched. The highest enrichment was cell process, regulation of major metabolic processes, intracellular part, intracellular organelle, membrane-bound organelle and egg white matter binding (Figures 4B–D). The significant enrichment results of the three GO categories are listed in Supplementary Table 3. According to the statistical results of the GO functional enrichment analysis (Supplementary Table 3), we identified 29 terms of 60 different parental genes related to muscle growth and development. See Supplementary Table 4 for details. According to statistics of the enrichment degrees of the 60 DE parental genes in each term (Figure 5A), it is found that the genes with higher enrichment degrees were gene 1570 (TTN), gene 23041 (MYBPC2), gene 6914 (MYBPC1), gene 1832 (NEB), and gene e22584 (MYH15), while the genes with lower enrichment degrees were gene 7152 (ZBCC9), gene 5917 (ACTR3b), and gene 5819 (EZH2), all of which are involved in the process of muscle growth and development, but the enrichment degree was highly different.

**FIGURE 6:**
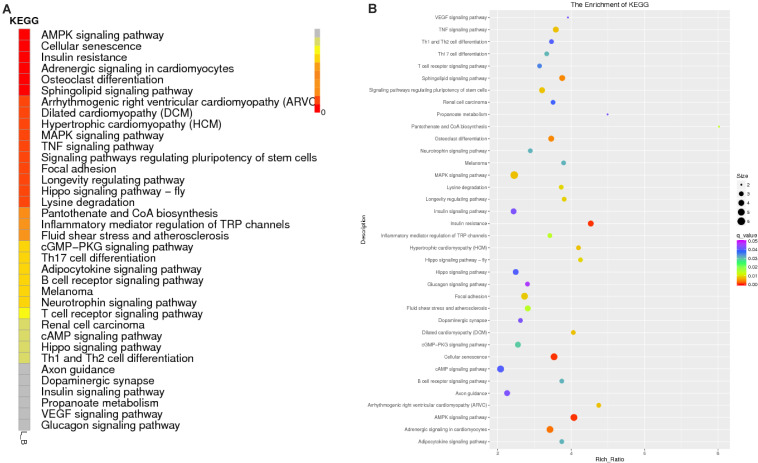
Enrichment results of the KEGG analysis. **(A)** Enrichment path *Q*-value distribution map, in which the vertical coordinate is the KEGG pathway entry, horizontal coordinate is the name of the sample, and different colors represent different enrichment degrees; **(B)** KEGG enrichment *Q*-value results for single groups, whereby each point represents the enrichment degree of the KEGG entry, and the closer the color is to red, the higher the enrichment degree. The size of each point indicates the number of genes enriched in the KEGG entry, and the larger the point is, the more genes enriched in the KEGG entry, and vice versa.

To predict the functions of significantly enriched parental genes, pathway analysis was conducted based on the KEGG pathway database. Among the 36 pathways with significant enrichment, the AMPK signaling pathway, cellular pathway, and cellular pathway and adaptive signaling in cardiomyocytes were the most significantly enriched (Figure 6A and Supplementary Table 6). All the samples were enriched by KOBAS, and the distribution map was made according to the significance of the *Q*-values of the KOBAS enrichment analysis (Figure 6B). Based on the above enrichment results, we identified 15 pathways (AMPK signaling pathway, adaptive signaling in cardiometrics, osteoblast differentiation, differentiated cardiopathy (DCM), hypertonic cardiopathy (HCM), MAPK signaling pathway, TNF signaling pathway, focal adhesion, and cAMP signaling pathway. These pathways were enriched for 49 parental genes (Table 4). One parental gene was involved in the regulation of multiple pathways. According to the statistics of the pathway enrichment analysis of the 49 parental genes (Figure 5B), the genes with higher enrichment were gene 20196 (AKT3), gene 1084 (PIK3CB), gene 25426 (PIK3R1), gene 31865 (MAPK8), and gene 21361 (MYL2), and the genes with lower enrichment were gene 6914 (MYBPC1), gene 7074 (BNM1L), gene 8206 (TBC1D1), and gene 822 (MECOM), all of which are involved in muscle processes. The pathways related to meat growth and development were also regulated, but the degree of enrichment was different.

**TABLE 4:** List of significantly enriched KEGG pathways related to muscle development.

KEGG pathway name	Map	Candidate_gene	Count
AMPK signaling pathway	map 04152	gene 1084; gene 1117; gene 20196; gene 21519; gene 23708; gene 25426; gene 33995; gene 5046; gene 5262; gene 5909; gene 8206; gene 8658	12
Adrenergic signaling in cardiomyocytes	map 04261	gene 1117; gene 13101; gene 13654; gene 15015; gene 20196; gene 21361; gene 22584; gene 27250; gene 27526; gene 5262; gene 5380; gene 8658	12
Osteoclast differentiation	map 04380	gene 1084; gene 11438; gene 12516; gene 17507; gene 20196; gene 22083; gene 1832; gene 25426; gene 27157; gene 31865; gene 8026	11
Dilated cardiomyopathy (DCM)	map 05414	gene 13654; gene 1570; gene 21361; gene 22584; gene 24411; gene 27250; gene 27380; gene 34158	8
Hypertrophic cardiomyopathy (HCM)	map 05410	gene 13654; gene 1570; gene 21361; gene 22584; gene 24411; gene 27250; gene 27380; gene 34158	8
MAPK signaling pathway	map 04010	gene 10636; gene 11438; gene 14563; gene 18172; gene 18729; gene 20196; gene 6914; gene 25232; gene 27250; gene 31330; gene 31865; gene 34246; gene 5152; gene 8026; gene 1832	15
TNF signaling pathway	map 04668	gene 1084; gene 17183; gene 20196; gene 25232; gene 25426; gene 31865; gene 5262; gene 7074; gene 8026	9
Focal adhesion	map 04510	gene 1084; gene 12407; gene 15015; gene 15498; gene 20196; gene 21361; gene 25426; gene 28102; gene 30957; gene 31865; gene 5152; gene 6914	12
cAMP signaling pathway	map 04024	gene 1084; gene 1832; gene 15015; gene 18729; gene 20196; gene 22584; gene 25426; gene 27526; gene 31865; gene 34652; gene 5262; gene 5380	12
Regulation of actin cytoskeleton	map 04810	gene 1832; gene 15015; gene 18729; gene 21361; gene 6914; gene 25426; gene 26115; gene 30957; gene 31330	9
Regulation of lipolysis in adipocytes	map 04923	gene 1084; gene 20196; gene 25426	3
mTOR signaling pathway	map 04150	gene 1084; gene 20196; gene 25426; gene 34246; gene 5909	5
Wnt signaling pathway	map 04310	gene 11438; gene 17507; gene 31865; gene 5380	4
PPAR signaling pathway	map 03320	gene 6914; gene 5046	2
Vascular smooth muscle contraction	map 04270	gene 15015; gene 6914	2

### Target miRNAs of Differentially Expressed circRNAs in Different Breeds of Beef Cattle

**FIGURE 7:**
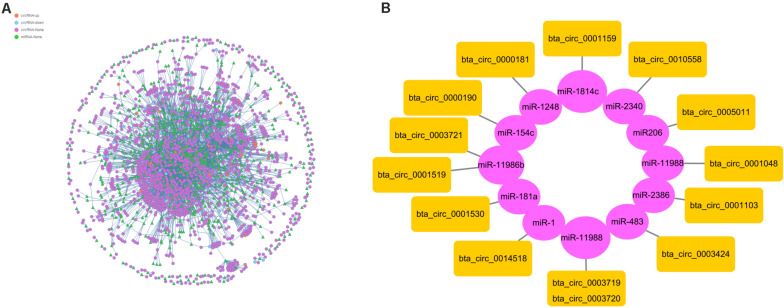
Interaction between circRNAs and miRNAs. **(A)** The circle nodes represent circRNAs, and the triangle nodes represent miRNAs. The lines represent the relationship between circRNAs and miRNAs. **(B)** Prediction of the target relationships between miRNAs and circRNAs.

To further understand the function of circRNAs, we used miRanda (see text footnote 4) to predict the interactions between circRNAs and miRNAs. According to the interaction data and differential expression of miRNAs and circRNAs, the interaction network data files were generated and imported into Cytoscape software. The attributes of the target circRNAs were visualized in the network, and the topological attributes of some networks are marked (Figure 7A). A total of 1799 circRNAs and 652 miRNAs were obtained to generate 5037 pairs of interactions between miRNAs and circRNAs (Supplementary Table 6), in which circ0013807 interacts with 37 miRNAs, circ0006152 interacts with 27 miRNAs, miR-11988 interacts with 449 circRNAs, and miR-11986b interacts with 376 circRNAs. According to the above differential expression and GO/KEGG/PPI enrichment analyses, we identified seven genes of different sources related to muscle growth, corresponding to 14 circRNAs and 11 target miRNAs (Figure 7B). It was found that there are multiple binding sites of miRNAs in some circRNAs (such as MYBPC1 and miR-11986b, RYR1 and miR-1) sequences. After a miRNA is adsorbed, it cannot regulate its corresponding target gene, thus a circRNA acts as a miRNA molecular sponge.

### Confirmation of circRNA Expression by qRT-PCR

**FIGURE 8:**
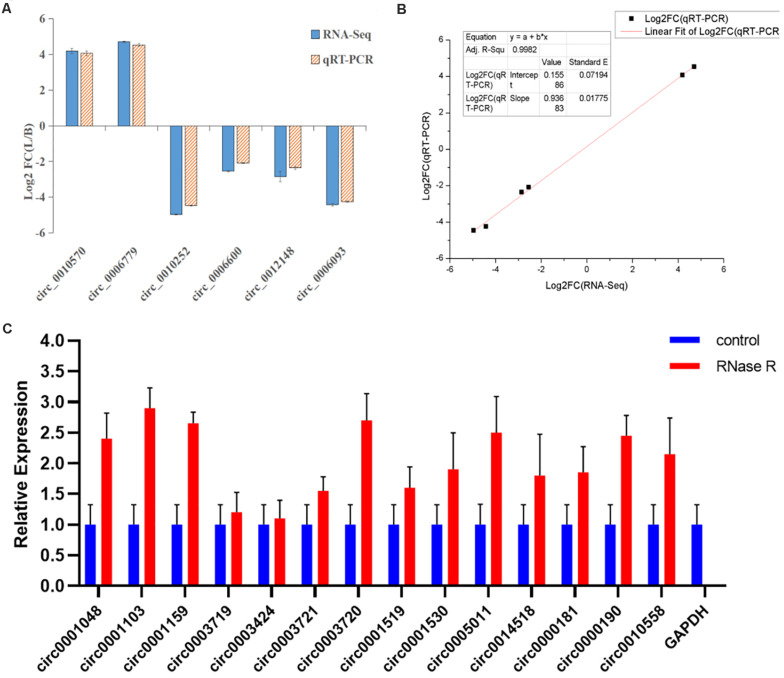
Linear fitting of RNA-seq and qRT-PCR circRNA expression data. **(A)** The circRNAs related to muscle growth and development were selected for qRT-PCR analysis. **(B)** The six selected circRNAs showed consistent results between the RNA-seq and qRT-PCR analyses, with a correlation coefficient (*R*^2^) = 0.9982. **(C)** After RNase R treatment, the expression level of circRNAs and GAPDH were measured. The *X*-axis indicates circRNAs, and the *Y*-axis indicates the relative expression level of circRNAs and GAPDH. Error bars indicates ±SD.

To verify the expression level of differentially expressed circRNAs, we randomly selected six highly expressed circRNAs and detected their expression level by qRT-PCR (Supplementary Table 7). These results were consistent with the trends observed in RNA-seq data (Figure 8A), with a correlation coefficient *R*^2^ = 0.9982 indicating that the RNA-seq results were reliable (Figure 8B). In this study, the cDNA generated by RNA reverse transcription was collected again after RNase R digestion, and the presence of circRNA was still detected in subsequent experiments. qRT-PCR quantitative detection results (Figure 8C) showed that compared with the control group (GAPDH), the expression of circRNA in RNase R digestion group was increased, and the expression of GAPDH was almost undetectable after digestion by exonuclease.

### Detection of Luciferase Activity of circ0014518 and bta-miR-1

**FIGURE 9:**
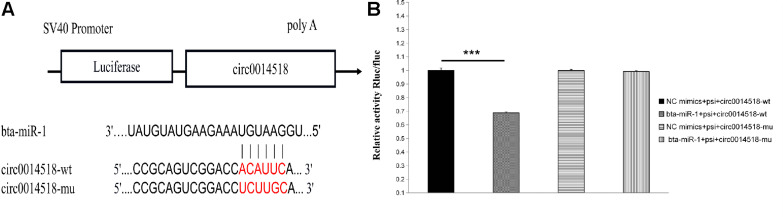
The prediction and verification binding site of circ0014518 and bta-miR-1. **(A)** The predicted binding site of between circ0014518 and bta-miR-1. **(B)** Verification binding site of circ0014518 and bta-miR-1 the luciferase reporter assay. ***P < 0.01.

The miRNA binding site of circ0014518 was screened according to the sequencing data and bioinformatics analysis results. It was found that circ0014518 contained bta-miR-1 binding site. Both RNA22 and RNAhybird predicted that circ0014518 contained bta-miR-1 binding sites (Figure 9A). In order to further verify the binding site of circ0014518 and miR-1, the psi-circ0014518 vector containing bta-miR-1 binding site was constructed, and the binding site was mutated from acauuc to ucuugc by site directed mutagenesis kit. Double luciferase reporter gene system was used to verify the binding site of circ0014518 and bta-miR-1. The results (Figure 9B) showed that the luciferase activity of bta-miR-1 and psi-circ0014518 was significantly lower than that of the control group (*P* < 0.01), but the luciferase activity of the mutant vector and bta-miR-1 co-transfected group was not significantly different from that of the control group, indicating that circ0014518 has a bta-miR-1 binding site.

## Discussion

In this study, we first examined the apparent differences in muscle fibers of different breeds of beef cattle. The results showed that there were obvious differences in the apparent observation of the muscle fibers of HE stained paraffin sections of the longissimus dorsi of Shandong black cattle and Luxi cattle. The length of a single muscle fiber of Shandong black cattle was significantly longer than that of Luxi cattle, and the number of nuclei in each muscle fiber was also greater. The border between the muscle fibers of Shandong black cattle was clearer and rounder than that of Luxi cattle. IPP software analysis showed that there were significant differences in the diameter and length of muscle fibers (*P* < 0.05) but no significant differences in the number, density or area of other muscle fibers (*P* > 0.05). The occurrence of these differences may be the key factors leading to the differences in meat production performance and meat quality of the two breeds of cattle after birth, which was also the research basis of this study to explore the underlying molecular regulatory mechanism.

We used RNA-seq technology to study the expression of circRNA in the longissimus dorsi muscle of different breeds of beef cattle. A total of 14,640 circRNAs and 4201 parental genes were detected. circ0013465 (UBE2D1), circ0011592 (UBE3A), and circ0001880 (MYL1) were the most highly expressed in the two libraries. PROSITE-ProRule annotation of these proteins (UBE2D1 and UBE3A) showed that they are involved in protein ubiquitination, which is part of the protein modifications that regulate cell metabolism within eukaryotes (Polge et al., 2016). MYL1 is a crucial protein for adequate skeletal muscle function and belongs to the myosin family (Ravenscroft et al., 2018). The ubiquitin proteasome system (UPS) is mainly responsible for the increased protein breakdown observed in muscle wasting. The Ube family of E3 ligases is a class of enzymes (i.e., troponin I, myosin heavy chains, and actin) that can guide the degradation of major contractile proteins. Their catalytic activity depends on the covalent binding of polyubiquitin chains catalyzed by a specific E2 on the substrate (Uhler et al., 2014). Studies have shown that UPS can control almost any muscle mass and recovery process in catabolism. The muscle-specific E3 ligase UBE family participates in the targeting of actin, myosin, troponin, and other major contractile proteins (Ravenscroft et al., 2018), indicating that the high expression of circRNAs plays a certain role in muscle development and redifferentiation.

According to the fold change >1.5 and *P* < 0.05 criteria, 655 differentially expressed circRNAs were identified, corresponding to 467 parental genes, 267 of which were upregulated and 388 downregulated in Luxi cattle. The function of a circRNA is reflected in its parental gene. Because there is no information about the annotation of circRNAs at present, we annotated the parental genes of differentially expressed circRNAs. As a result, is the genes were annotated in 65 different GO terms, which mainly play a role in biological processes such as regulation of cell process, regulation of metabolic process and part in the cell. We identified 29 related terms. There were 60 different genes associated with muscle growth and development. The genes with higher enrichment were gene 1570 (TTN), gene 23041 (MYBPC2), gene 6914(MYBPC1), gene 1832 (NEB), and gene 22584 (MYH15). All of the above genes participate in muscle growth and development, and their corresponding circRNAs also play a role in this process. The number of circRNA parental genes in the different samples was significantly different. The difference reflects the cumulative effect of circRNAs on expression characteristics. Based on the KEGG pathway database, we further analyzed the circRNAs and found that the AMPK signaling pathway, cellular signaling pathway, and cellular signaling and alternative signaling in cardiomyocytes were the most significantly enriched pathways. In our study, based on the above enrichment results, we identified 15 distinct enriched pathways related to muscle growth and development, including the AMPK signaling pathway, MAPK signaling pathway and adaptive signaling in cardiometrics, which influence muscle fiber processes (O’Connell et al., 2013; Zeng, 2016); the MTOR signaling pathway and Wnt signaling pathway, which are involved in the regulation of skeletal muscle development and regeneration (Fernández-Martos et al., 2011; Hong-Bo et al., 2017; Cong, 2018); the PPAR signaling pathway, which is involved in the regulation of intramuscular fat deposition (Li, 2018) and the cytoskeletal signaling pathway. According to the statistical data, the genes with higher enrichment levels were gene 20196 (AKT3), gene 1084 (PIK3CB), gene 25426 (PIK3R1), gene 31865 (MAPK8), gene 21361 (MYL2), and gene 6914 (MYBPC1), which could indicate that the circRNAs produced by these genes may play a role in the growth and development of muscle through these pathways. Combining these results with the above results, we identified seven parental genes (TNN, MYBPC1, NEB, MEF2C, MYH7, PPP2R3A, and RYR1) and 38 corresponding circRNAs. Comparing these results with previous research results, significant differences were observed in the expression of circRNAs related to the muscle development of different breeds of cattle, suggesting that circRNAs may play an important role in muscle development. Whether these circRNAs have specific functions and what the functional mechanism is need to be studied further.

circRNAs can play important roles by regulating the transcription and expression of their parental genes (Zheng et al., 2017). At present, there is a relatively limited understanding of the details of the formation of circRNA and its functional mechanism. circRNA can be obtained by transcription of protein-coding genes or intergenic regions (Qu et al., 2015). The formation of circRNA from a protein-coding gene is caused by the variable splicing of the parental gene (Ashwal-Fluss et al., 2014). Therefore, there should be a certain correlation between a circRNA and its parental gene expression. We found that one source gene may produce multiple circRNA subtypes. For example, the MYBPC1 gene can produce nine different circRNA subtypes. We obtained the FPKM value of the two varieties and found that both were differentially expressed. Although one source gene may produce multiple circRNA subtypes at the same time, only 3 or 4 of them have high expression levels, and the rest have low expression levels, which indicates that the cyclization of RNA in muscle is strictly regulated. To further understand the biological function and molecular function of the parental genes of significantly differentially expressed circRNAs, we predicted the interaction between circRNAs and miRNAs and constructed a network from the interaction data. The interaction network showed that a single miRNA may be correlated with multiple differentially expressed circRNAs, and there have been reports that circRNAs can competitively adsorb miRNAs (Zheng et al., 2016). Based on the high-throughput sequencing results, we selected 14 circRNAs related to muscle development as candidate circRNAs (circ0001048, circ0001103, circ0001159, circ0003719, circ0003424, circ0003721, circ0003720, circ0001519, circ0001530, circ0005011, circ0014518, circ0000181, circ0000190, circ0010558). In addition, the target miRNAs were predicted, the corresponding mRNA targets of the miRNAs were predicted, and the circRNA-miRNA-mRNA network was constructed to further study the regulation of muscle development. We will further verify this network in future experiments, which provides a new basis for the study of muscle development in cattle.

In the study, the 14 circRNAs quantitatively detected were required to identify whether they were real circRNAs. The results showed that circRNA could resist RNase R digestion, but linear RNA could not. Therefore, the candidate circRNAs (circ0001048, circ0001103, circ0001159, circ0003719, circ0003424, circ0003721, circ0003720, circ0001519, circ0001530, circ0005011, circ0014518, circ0000181, circ0000190, circ0010558) are circular RNAs. At the same time, a circRNA was screened for functional verification. Luciferase reporter gene system was used to verify the binding site of miR-1 in circ0014518, indicating that circ0014518 can be used as a molecular sponge of miR-1. It has been reported that miR-1 participates in the regulation of skeletal muscle development through target gene Pax7. We intend to further verify this in future experiments. In addition to the above findings, there were pathways that were not found in our study that are known to be important and enriched for many parental genes and some that have been reported in previous studies to regulate muscle growth and development. The miRNAs that have been shown to be expressed specifically or preferentially in muscles are called muscle-specific miRNAs (muscle-specific microRNAs, myomiRs) (Horak et al., 2016) and include miR-1, miR-206, miR-128, miR-483, miR-2425-5p, miR-181a, miR-208a, miR-208b, and miR-486 (Sempere et al., 2004; Van Rooij et al., 2009; Small et al., 2010). MiR-206 is specifically expressed only in skeletal muscle, and its targeted circRNAs (circ0001651, circ0010874, circ0010882, circ0010890, circ0010896, circ0012793) were not significantly differentially expressed. Whether these circRNAs have specific functions and what their functional mechanisms are need further study.

## Conclusion

In this study, we investigated the expression of circRNAs in muscle tissues of different breeds of beef cattle, obtained 655 differentially expressed circRNAs and 467 parental genes, selected 15 circRNAs related to muscle development as candidate circRNAs, and predicted the target miRNAs. These findings may provide clues for further research on muscle development in different breeds of beef cattle.

## Data Availability Statement

We have stored the RNA sequence data in the public domain GEO NCBI, GEO accession numbers: SRR11819095, SRR11819094, SRR11819093, SRR11819092, SRR11819091, and SRR11819090.

## Ethics Statement

The animal study was reviewed and approved by the Animal Experiments of Qingdao Agricultural University IACUC (Institutional Animal Care and Use Committee).

## Author Contributions

YD, XB, CX, and RL designed the study. RL and XL conducted the experiment. RL, XL, and XB performed and collected the data. RL analyzed the data and wrote the manuscript. All authors read and approved the final manuscript.

## Conflict of Interest

The authors declare that the research was conducted in the absence of any commercial or financial relationships that could be construed as a potential conflict of interest.

## Footnotes

1 http://www.usadellab.org/cms/index.php?page=Trimmomatic

2 http://tophat.cbcb.umd.edu/

3 https://www.ncbi.nlm.nih.gov/genome/82?genomeassemblyID=371813

4 http://www.microrna.org/microrna/home.do

5 https://pypi.org/project/goatools/

## Abbreviations

“HE staining”: hematoxylin and eosin staining
GO: Gene Ontology
KEGG: Kyoto Encyclopedia of Genes and Genomes
PPI: Protein-protein interaction networks
SRPBM: spliced reads per billion mapping
RNA-seq: RNA sequencing technology
qRT-PCR: real-time quantitative PCR
SPONG: the interaction network between miRNA and circRNA.
